# Immunosuppressive therapy in children with primary nephrotic syndrome: single center experience, Karachi, Pakistan

**DOI:** 10.1186/s12882-019-1347-5

**Published:** 2019-07-03

**Authors:** Khemchand Netaram Moorani, Harnam Moolchand Hotchandani, Aasia Mohammad Zubair, Neelesh Chander Lohana, Nanga Ram Veerwani

**Affiliations:** 1 Department of Pediatric Nephrology, National Institute of Child Health (NICH), Jinnah Sindh Medical University, Karachi, Pakistan; 2 Department of Pediatric Nephrology, The Kidney Center Postgraduate Training Institute (TKC-PGTI), Karachi, Sindh 75500 Pakistan

**Keywords:** Nephrotic syndrome, Minimal change disease, Oral prednisolone, Levamisole, Cyclophosphamide, Cyclosporin, Mycophenolate mofitil

## Abstract

**Background:**

Majority of children with nephrotic syndrome are steroid sensitive, but treatment of difficult to treat nephrotic (frequent relapsing, steroid dependent and steroid resistant) syndrome is challenging. Low dose steroid, levamisole, cyclophosphamide (CPM), mycophenolate mofetil (MMF) and calcineurin inhibitors (CNIs) are the common options of treatment.

Objective of the study was to determine the response to steroid and alternative immunosuppressive agents (ISAs) in children with difficult nephrotic syndrome (DNS).

**Methods:**

This is a retrospective cohort study of 176 children with DNS, managed over 12 years at The Kidney Center-Postgraduate Training Institute, Karachi- Pakistan from 2005 to 2017.

Initial episode was treated with daily oral prednisolone (OP) for 4–8 weeks followed by alternate day OP for 12–24 weeks. Subsequently low dose OP, levamisole (Leva)and cyclophosphamide was used for frequent relapsing (FR)/ steroid dependent (SD). All with initial steroid resistance and non- responders to leva and or cyclophosphamide were biopsied and treated with CNIs and MMF. Data was analyzed using descriptive statistics.

**Results:**

There were 130(73.86%) children with FR/SD and 46(26.13%) with SRNS. All children with SR (46) and 86 with FR/SD were biopsied. Minimal change disease (60.60%) and focal segmental glomerulosclerosis (FSGS 23%) were the two common lesions. Majority (73.86%) received single OP whereas divided doses were administered in 26.13% cases. Daily OP was used for 4, 6 and 8 weeks in 61.36,28.4 and10.22% respectively. Steroids were tapered over 3 (31.81%),4 (52.27%) and 6 months (15.90%). Levamisole, CPM, cyclosporin (CS) and MMF were used sequentially in 45, 54.23, 50 and 20% respectively. Combination of MMF and CS was used in 11.29% of cases.

Levamisole was effective in 80%, CPM induced complete remission (CR, 57.77%) or partial remission (PR, 22.22%), CS induced CR 46.59% and PR 39.77%. MMF showed PR and CR 69 and 12.82% respectively. At last follow up, 46% were maintaining remission while off treatment, whereas 35% are maintaining remission on therapy,10.23% lost- to-follow, 5.68% progressed to chronic kidney disease. Mortality was 2.84% and it was due to infection and uremia.

**Conclusion:**

Majority had steroid sensitive MCD. Levamisole and cyclophosphamide were effective in maintaining remission in FR/ SD. FSGS was responsible for resistance to steroid and alternative ISAs. Cyclosporin was effective in inducing remission in SRNS. Mortality was less than 3%.

## Background

Nephrotic syndrome (NS) is a chronic relapsing disease with good long-term outcome. There are lot of regional practice variations in the treatment of nephrotic syndrome. Historically, International Study of Kidney Disease in Children (ISKDC) in early 70s recommended daily oral steroid 60 mg/m^2^ for 4 weeks followed by 40 mg /m^2^ on alternate day for 4 weeks [1]. Subsequently use of high dose steroid (60 m^2^/day) for 6 weeks followed by 40 mg/m^2^ on alternate day for 6 weeks showed reduction in frequency of relapses and steroid dependency [2]. Kidney Disease Initiative Global Outcome (KDIGO) guidelines (2012) also suggested same dose with more flexibility of using oral prednisolone (OP) 60 mg/m^2^/day for 4–6 weeks followed by tapering over 2–5 months [3]. However, more recent randomized controlled trials have shown that short course (2–3 months) treatment with OP is neither inferior to long course (4–6 months) nor associated with increased risk of frequent relapses and steroid dependency [4–6]. More than 85–90% of children are initial steroid sensitive and achieve remission within 4–6 weeks and 10–15% behave as initial steroid resistant (SR) [1, 3, 7].

About 60–80% of steroid responder develop relapses and 40–60% will become frequent relapser (FR) and 30% become steroid dependent (SD). Patients with FR, SD and SR are known as difficult nephrotic syndrome (DNS) since these require alternate immunosuppressive strategies to avoid steroid toxicity, severe infections, hypertension and acute kidney injury or chronic kidney disease (CKD). That is why difficult nephrotic syndrome with recurrent or persistent nephrotic range proteinuria has been considered as CKD [3, 7, 8].

Management of DNS is challenging. There are multiple strategies for management of children with FR/SDNS, including use of low dose OP on alternate day for prolonged duration, use of levamisole (LEVA) along with alternate day low dose OP, cyclophosphamide (CPM) for 2–3 months, mycophenolate mofetil (MMF) and calcineurin inhibitors (CNIs) like cyclosporine or tacrolimus [9–12]. CNIs are the treatment of choice for SRNS and MMF is an alternative if serum creatinine is raised [3, 7, 8, 12]. Rituximab (anti-CD 20) has been the last sword for difficult SD and rarely used in children with SR [13]. Children with SR and SD should undergo renal biopsy for diagnosis, before starting CNIs and to assign long term prognosis; the two most common biopsy findings are minimal change disease (MCD) in 85% of steroid sensitive and focal segmental glomerulosclerosis (FSGS) in SRNS [14, 15].

There are various studies from developing countries on different aspects of nephrotic syndrome but there was no study from Pakistan in pediatric population from a single center on treatment outcome of children with difficult nephrotic syndrome [16, 17]. Current study highlights the long-term experience of treating children with DNS using various immunosuppressive agents (ISAs) at Tertiary Care Center, over 12 years period from Karachi, Pakistan. The objective of the study was to determine response to OP in initial episode of NS and to alternate immunosuppressive agents (ISAs) in children with FR/SD and SRNS.

## Methods

This was a retrospective cohort comprising of 176 patients with DNS who were treated sequentially with ISAs over 12 years from 2005 to 2017 at The Kidney Center-Postgraduate Training Institute (TKC-PGTI), Karachi- Pakistan. TKC-PGTI is a private tertiary care center with facilities of nephrology, urology and dialysis services with more than 50% welfare support**.** Institutional ethical approval was taken and consent from individual patient or family was not required.

**Fig. 1:**
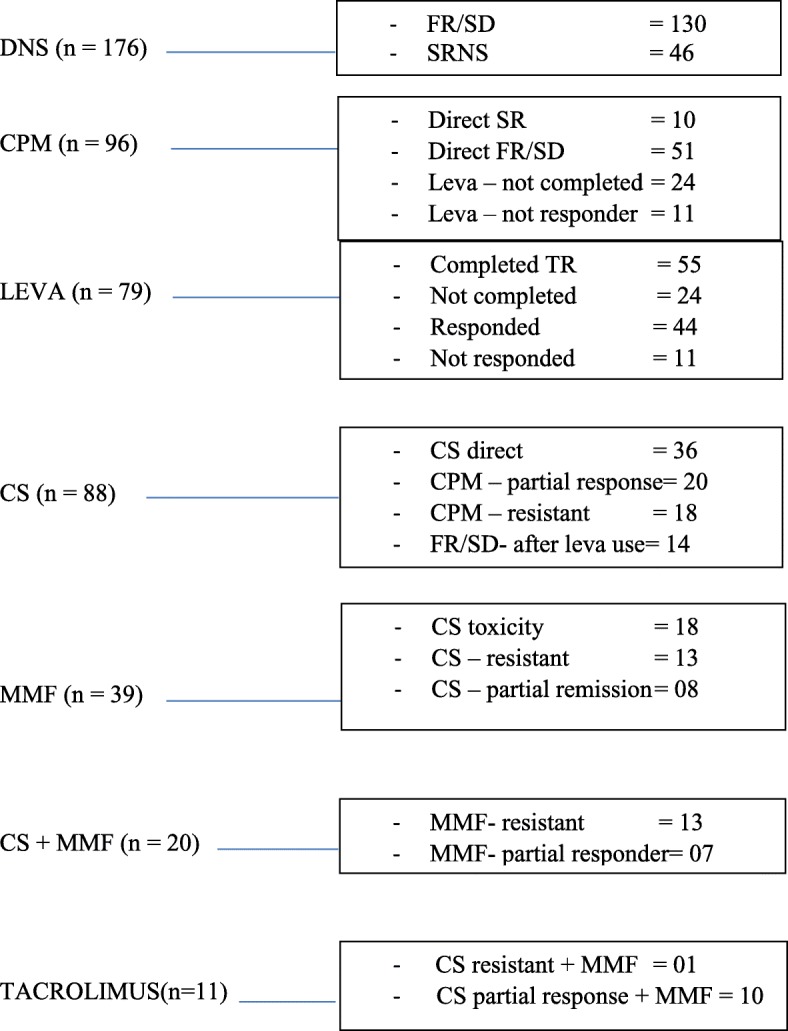
Flow chart of Sequential Immunosuppressive Therapy in Children with Difficult Nephrotic Syndrome

All children with first episode were treated with OP at 60 mg/m^2^/day for 4–6 weeks followed by slow tapering over 3–6 months. Initial two relapses were treated like first episode. Further categorization was done according to number of relapses over 6–12 months and need of high dose steroid, into infrequent relapser, FR, SD or SR and managed with sequential immunosuppressive agents as defined in operational definitions and according to flowchart (Fig. 1 Flow chart of Sequential Immunosuppressive Agents in DNS) [1–3]. Children who behaved as infrequent relapsers were excluded from analysis.

Patients with FR/ SD were treated initially with LEVA 2.5 mg/kg on alternate day in two divided doses during early years and with single daily dose 2–2.5 mg/kg for 6–24 months along with low dose alternate day OP (0.25–0.5 mg/kg). If no response to LEVA, then CPM 2–3 mg/kg/day for 2–3 months with a cumulative dose of 168 mg/kg/course after initial remission with daily steroid [3]. If patients still behaved as FR/SD after use of CPM, then biopsied, and placed on either CS or MMF depending up on renal functions and drug toxicity.

All patients with initial SR were biopsied and treated according to histopathological diagnosis**.** MCD in SRNS was also treated with cyclophosphamide in early years due to cost issues. However, CS was used as first choice CNI, in a dose of 5 mg/kg/day in two divided doses with monitoring of clinical edema, spot urine protein creatinine ratio (suPCR) and serum creatinine (Cr) for 12 months in SDNS and then 3 mg/kg/day as maintenance dose for further 12–36 months in CS responders. Dose was reduced by 25% if serum Cr increased above 1 mg/dl or stopped if Cr remained high after one week of reduction of CS dose. Tacrolimus and MMF were used in cases who developed cosmetic CS-toxicity or resistant to CS respectively. MMF and CS were combined if either drug alone was not effective after 3–6 months. Complete blood counts and serum Cr were monitored for MMF and CS/ Tacrolimus toxicity respectively. Response to ISAs was assessed by clinical edema and spot urine protein creatinine ratio (suPCR) and categorized as defined in operational definition [1–3].

Patients were followed - up by single pediatric nephrologist and his team.

Data including demographics, details of initial steroid therapy, type of NS according to steroid response (SD or SR), indications and outcome of biopsy, response to various ISAs and major adverse effects were collected from hospital case record and analyzed on SPSS-16. Qualitative variables like gender, type of NS and treatment outcome were represented by frequencies and percentages whereas quantitative variables like age were represented by mean ± standard deviation.

Operational Definitions [1–3].

Nephrotic Syndrome: Combination of clinical edema, nephrotic range proteinuria (suPCR ≥2 or 3_+_ protein on dipstick), hypoproteinemia (< 5.5G/dl), hypoalbuminemia (< 2.5G/dl) and hypercholesterolemia (> 250 mg/dl).

Frequent Relapser: Two or more than two relapses in 6 months or more than 4 in a 12 months period.

SDNS: Two consecutive relapses on steroid therapy or occurred within 14 days of switching to alternate day prednisolone.

SRNS: Persistence of edema and or proteinuria (suPCR > 2) after 4–6 weeks of OP 60 mg /m^2^ / day.

Complete remission (CR): Disappearance of edema and proteinuria (suPCR < 0.2)/urine dipstick nil or < 1_+_.

Partial remission (PR): disappearance of edema but persistence of non-nephrotic range proteinuria (suPCR 0.2- < 2).

Difficult nephrotic syndrome (DNS): Frequent relapsing, SD and SR were considered as DNS.

CPM resistant NS: Persistent nephrotic range proteinuria and edema after 8–12 weeks of 2-3 mg/kg/day of CPM.

CS resistant NS: Persistent nephrotic range proteinuria and edema after 3 months of 5 mg/kg/day of cyclosporin.

Levamisole response: Effective if < 1 relapse in 12 months on LEVA treatment and not effective if child had ≥ two relapses within first 6 months or ≥ three relapses in 12 months period while on LEVA.

## Results

Our study cohort comprises of 176 patients with DNS (FR, SD and SR) who were managed sequentially with different ISAs after initial induction of remission with OP. Mean age was 4.78 ± 3.23 years. There were 100(56.81%) male and 76 female. Base line demographics, clinical, biochemical and urinary characteristics of study population are shown in Table 1.

**Table 1:** Baseline characteristics of children with difficult nephrotic syndrome

Anthropomeasurements of study population *n* = 176		
Variable	Mean ± SD	Min – Max
Age (years)	4.78 ± 3.23	1–15
Weight (kg)	17.97 ± 8.99	7.5–51
Height (cm)	98.68 ± 23.60	72–166
BSA (msq)	0.69 ± 0.25	0.4–1.8
Clinical presentation of children with difficult nephrotic syndrome		
Feature	N	%
male	100	56.81
Edema	174	98.86
Hypertension	16	9.09
Gross hematuria	5	2.84
Renal failure	2	1.14
Diagnostic biochemistry and urinary PCR in children with difficult nephrotic syndrome		
Variable	Mean ± SD	Min – Max
Total protein (g/dl)	4.53 ± 0.81	2.7–7.9
Serum albumin (g/dl)	2.00 ± 0.58	1–4.5
Serum cholesterol (mg/dl)	369.93 ± 114.25	5–730
Serum creatinine (mg/dl)	0.37 ± 0.30	0.06–2.30
Spot urine- PCR (mg/mg)	9.01 ± 6.52	0.2–39.5

*PCR* Protein Creatinine Ratio

This table shows that edema was the most common clinical presentation (98%) followed by hypertension (9%) and gross hematuria (2.8%). Biochemical parameters at initial presentation were hypoproteinemia (4.53 ± 0.81 g/dl), hypoalbuminemia (2.0 ± 0.58 g/dl), hypercholesterolemia (369.93 ± 114 mg/dl) and it was nephrotic range proteinuria (suPCR > 9 ± 6.52). Baseline renal functions were normal (serum Cr level = 0.37 ± 0.30 mg/dl) except in two cases.

Table 2 shows the pattern of initial OP treatment and subsequent course of study population. Majority of patients (73.86%) received single morning dose whereas 26% received divided doses. Daily OP was administered for 4,6 and 8 weeks in 61.36, 28.4 and 10% respectively. Tapering of steroid therapy on alternate day varied from 3 months (31.81%) to 6 months (15.9%).

**Table 2:** Pattern of corticosteroid therapy and subsequent outcome in difficult nephrotic syndrome (*n* = 176)

Parameter	Number	Percentage
Dose of initial oral steroid treatment		
Prednisolone (60 mg/m^2^/day)	176	100
Timing of daily oral prednisolone		
Single morning dose	130	73.86
Divided doses	46	26.13
Duration of daily prednisolone		
4 weeks	108	61.36
6 weeks	50	28.40
8 weeks	18	10.22
Total duration of initial prednisolone		
3 months	57	32.38
4 months	92	52.27
6 months	27	15.34
Categories based on steroid response and frequency of relapses		
Steroid sensitive	130	73.86
Frequent relapser	75	57.69
Steroid dependent	55	42.30
Steroid resistant	46	26.13
Steroid toxicity in 105 children with FR/SD and SRNS		
Cushingoid	81	46.02
Severe infection	18	10.22
Steroid psychosis	3	1.7
Hypertension	3	1.7

Table 2 also shows that majority of study population was steroid responsive (73.86%) whereas 26% were steroid resistant. Among the steroid responsive, 57.69% were frequent relapser and 42.3% were SD. Steroid induced cushingoid appearance and severe infections were the two major complications observed in 46 and 10% of cases respectively.

Table 3 shows the spectrum of histopathological diagnosis in children with DNS. Forty- six with SRNS and 86 with FR/SD underwent renal biopsy during the study period. MCD and FSGS were the most common histopathological diagnosis in FR/SD and SRNS; found in 79.06% and in 45.65%respectively. Over all, the most common histopathological diagnosis in 132 cases with DNS was MCD (60.60%) followed by FSGS (22.72%) and others (16.68%).

**Table 3:** Spectrum of histopathological diagnosis in 176 children with difficult nephrotic syndrome

Histopathological diagnosis	FR/SD *N* (%)	SRNS *N* (%)	Total *N* (%)
Number of patients (%)	130 (73.86)	46 (26.13)	176 (100)
Renal biopsy status			
- Done	86 (66.15)	46 (100)	132 (75)
- Not Done	44 (33.84)	00 (0)	44 (25)
Minimal change disease	68 (79.06)	12 (26.08)	80 (60.60)
FSGS	9 (10.46)	21 (45.65)	30 (22.72)
MPGN	3 (3.48)	7 (15.21)	10 (7.75)
MGN	3 (3.48)	6 (13.04)	9 (6.81)
IgM Nephropathy	3 (3.84)	0 (0)	3 (2.27)

Details of sequential immunosuppressive therapies and their outcome in DNS is shown in Table 4. Sequentially, LEVA, CPM, CS and MMF were used in 55(31.25%), 90(51.13%), CS 88(50%) and 39(22%) cases respectively. Combination of CS and MMF was used in 20 cases (11.29%) whereas tacrolimus with or without combination of MMF was used in 11 cases. Fifteen received two or more than two courses of CS.

**Table 4:** Outcome of sequential alternative immunosuppressive therapies in children with DNS

Response to levamisole in children with FR/SD(*n* = 55)			
Outcome *N* (%)	FR 41 (74.54%)	SD 14 (25.46)	Total 55 (100)
Effective	34	10	44 (80)
Not effective	7	4	11 (20)
Response to cyclophosphamide in children with DNS (*n* = 90)			
Outcome *N* (%)	FR/SD 70 (77.77)	SRNS 20 (22.22)	90 (100)
Complete remission	45	07	52 (57.77)
Partial remission	13	07	20 (22.22)
Resistant	12	06	18 (20)
Response to cyclosporin in children with DNS (*n* = 88)			
Outcome *N* (%)	FR/SD 49 (55.68)	SRNS 39 (44.31)	88 (100)
Complete remission	30	11	41 (46.59)
Partial remission	18	17	35 (39.72)
Resistant	1	11	12 (13.63)
Response to mycophenolate mofetil in children with DNS (*n* = 39)			
Outcome *N* (%)	FR/SD 21 (53.84)	SRNS 18 (46.15)	39 (100)
Complete remission	4	1	5 (12.82)
Partial remission	16	11	27 (69.23)
Resistant	7	6	13 (33.33)
Response to combination of CS and MMF in children with DNS (*n* = 20)			
Outcome *N* (%)	FR/SD 12 (60)	SRNS 8 (40)	20 (100)
Complete remission	4	2	6 (30)
Partial remission	7	5	12 (60)
Resistant	1	1	2 (10)
CS dependent^a^	9	6	15 (17.04)

^a^ ≥ −2courses

Levamisole was offered to 79 children with FR/SD but 24 could not complete 6 months so data of 55(FR/SD = 41/14) were analyzed. LEVA was effective in maintaining remission in 80% cases and more so in frequent relapsers. There was pancytopenia and allergic rashes each in one.

Cyclophosphamide was used in 96 children with DNS, but it was discontinued in 6 due to severe infections or bone marrow suppression (BMS). CPM induced CR in 57.77% and PR in 22.22% whereas 20% were resistant to CPM and 6 with initial SR (6/20) were also resistant to CPM. Adverse effects of CPM were severe infections including disseminated chicken pox (9), BMS (5), alopecia (3) and hyperpigmented nail beds (3).

Table 4 shows that CS induced CR and PR in 47 and 40% respectively. Fifteen (17%) were CS dependent since they required more than one course and 12(13.63%) were resistant to CS and majority were initial steroid resistant cases. Eleven (12.5%) patients required tacrolimus for CS -associated cosmetic toxicity. Main adverse effects were gum hyperplasia (5), hypertrichosis (6), renal dysfunction (7) and deafness (1).

MMF was effective in induction of PR in 69.23%, CR in 13 and 33% were resistant to MMF. Similarly, it induced PR in 60% when combined with CS. Enteric coated formulation was well tolerated.

Overall, outcome at last follow up (Table 5) showed that 81(46%) children were maintaining either CR (76) or PR (5) and are off treatment, whereas 62(35%) are maintaining remission on therapy. We lost to follow 18(10.23%) children,10 progressed to CKD/ESRD due to FSGS (5), MGN (2), MCD (2), MPGN (1) and one with MCD developed CS nephrotoxicy due to prolonged use for more than 12 months without monitoring. Mortality was observed in 2.84% and it was due to infection in 03 children with MCD and uremia each one secondary to MPGN and FSGS.

**Table 5:** Long-term outcome of immunosuppressive therapies in children with difficult nephrotic syndrome(*n* = 176)

Long-term outcome	FR/SD *N* = 130	SRNS *N* = 46	*N* (%)
Complete remission off treatment	65	11	76 (43.18)
Partial remission off treatment	0	5	5 (2.86)
Complete remission on treatment	34	8	42 (23.86)
Partial remission on treatment	12	8	20 (11.36)
CKD/ESRD	4	7	11 (6.25%)
FSGS	0	5	10 (5.68%)
MPGN	0	1	
MGN	2	0	
MCD	1	1	
MCD _+_ CS-Toxicity	1	0	
Lost to follow	10	8	18 (10.23)
Expired			5 (2.84%)
Uremia	0	2	
Infection	2	1	

## Discussion

This is a first long-term study in a single center describing the personal practice variation in the management of difficult nephrotic syndrome in children over more than 12 years (2005–2017) from Pakistan. This study describes the various aspects of management of DNS including the clinical and biochemical characteristics, varying steroid protocols (dosage, timing and schedule of administration, duration of daily and alternate day steroid therapy), patient’s initial and subsequent behavior to steroids and frequency of relapses based out come in the form of either FR/SD or steroid resistance at initial or later disease course. In addition, the spectrum of histopathological diagnosis in 132 children with DNS and use of alternative immunosuppressive therapies, associated adverse events and long-term outcome including mortality was looked vigorously.

Our results show that majority of patients (73.86%) were initial steroid sensitive which ultimately behaved as FR/SD requiring sequential multi-strategic therapies and 26.13% of patients were either early or late SR. This is consistent with the report from ISKDC and KDIGO [1, 3]. Little higher frequency of steroid sensitivity (> 90%) has been reported in different studies using different duration of steroid therapy [8, 12]. This may be explained on basis of high prevalence of intercurrent infections or genetic and racial variation which may affect the response since in our study, there was lot of ethnicity and linguistic variation. Though, these socioeconomic, nutritional and prevalent infections were not investigated, but authors assume that these were important factors affecting practice variation and outcome.

Though, in majority (73.86%) of children, initial episode was treated with single morning dose of OP but significant number (26%) also received divided dosage regimen (Table 2). This divided dosage practice was in earlier period of study and in later period it become common and standard practice to use single morning dose. Similarly, duration of initial daily steroid and alternate day prednisolone also varied accordingly; suggesting the changing practice of treating nephrologist as well as diversity of patients with different linguistic and distant residential location as well as time and money  to travel, may have influenced the duration of steroid resulting in high frequency of steroid toxicity (46%). This is not different from the international literature which reveal that either 4–6 weeks daily steroid or use of methyl prednisolone or extending duration to 8 weeks for defining steroid resistance also contribute for steroid toxicity [12, 18]. Though, many guidelines have been developed more recently but due to lack of local guidelines, still practice variation exists considerably from time to time, center to center and depending upon experience of treating pediatrician and pediatric nephrologists [7, 17, 19].The most current evidence for treatment of initial episode suggest that OP therapy for 6 months is not superior to short course 2–3 months) with respect to reduction of the risk for relapse suggesting a significant change in standard practice [4–6, 8].

The spectrum of histopathological diagnosis in our study is consistent with local and international literature; that MCD has been the most common underlying biopsy in children with SSNS (> 80%) and FSGS in steroid resistant [14, 15].

Considering the management of DNS, sequentially, we used LEVA, CPM, CS and MMF in 31.25, 51.13, 50 and 22% cases respectively. This practice of sequential selection of various ISAs is more or less consistent with a recent report on use of ISAs in FR/SD children from Saudi Arabia in which LEVA, CPM, CS and MMF were used in 33.3,20, 21.6 and 25% respectively [17]. Since number of patients was too small in the quoted study and it was only in FR/SD so use of CPM and CS is much less than ours.

Levamisole, an immunomodulatory drug being focus of current research in FR and SD, was found to be effective in maintaining remission in almost 80% in FR/SD patients. Many recent studies including multicenter RCT have shown similar results (77% in FR/ SD [10, 18, 20–22]. Though most of studies have used alternate day levamisole but we have used as singe alternate or daily dose for more than 2 years without significant toxicity. It was more effective in FR than in SD as shown by others [10, 21].

Cyclophosphamide is commonly used steroid sparing agent effective in inducing remission in SS and in SRNS [3, 11, 12]. We found overall short-term CR and PR in 57.77 and 22.22% respectively in children with DNS. This is much lower than 97%(CR/PR) shown in a recent local study in which CPM was used in all steroid sensitive patients [17]. Similar to our results on short -term outcome have been reported in earlier metanalysis and guidelines [3, 11]. In this study, CPM was also effective in inducing short- term CR/PR in 32% of 22 steroid resistant children. We used CPM during earlier period of study, in patients who were either non-affording for CNIs and had MCD as histopathological diagnosis or in children with raised creatinine. This is significant in developing countries since it may save the cost and delay the use of CNIs which may be a significant risk factor for long term irreversible nephrotoxicity and CKD. Since patients were biopsied before initiating CPM so all patients with MCD received CPM before CNIs. Earlier studies had shown similar percentage of response in SRNS that it may induce CR or PR in 30%-40 of SRNS [11, 12, 17, 18]. However, a most recent study has reported a higher rate of sustained remission than ours and low rate of relapse (11.2%) with use of CPM in 62 children compared to CS (relapse rate 6.2%) in 65 patients. However, the cummulative dosage of CPM in such long duration (3-6 months) use can not be compared with commonly used cummulative dose of 168 mg/course [23]. Major adverse effects in our study were severe infections (10%), BMS (5.5%) and alopecia (3.3%) are lower than reported by Lata K et al. [11]

CNIs are the most commonly used immunosuppressive agents both in FR/SD and SR and have been recommended by KDIGO as first line treatment in SRNS.CNIs are found to be effective in induction and maintenance of CR or PR remission in 40–80%, more so in FR/SD [3, 23–25]. We found that CS was effective in inducing CR and PR in 61 and 36.73% respectively in 49 children with FR/SD. Similar response rate has been reported in recent studies [24–26].CS induced CR and PR in 28 and 43.5% in our 46 children with SR. Similar response (41%) has been documented by us in FR/SD and SR-FSGS and in a recent study from China [16, 23, 26]. However, in another study comparing CPM with CS in SRNS, higher success rates (70.8%) compared to CPM (51.6%) has been reported and more so in children with MCD [18]. Adverse effects of CNIs in our study were gum hyperplasia(5.6%), hypertrichosis (6.8%), renal dysfunction (8%) and deafness(1%) are less than reported in the literature [7, 16]. However, one patient developed ESRD after CS use without monitoring.

Mycophenolate was used in children who were either CS resistant or has impaired renal functions or developed cosmetic toxicity after CS use. When used alone, MMF induced PR in 69.23% and CR in 12.8% of children with DNS. It was noted that MMF was more effective in FR/SD rather than SRNS (95% vs 66%) but not better than CS. More or less similar results have been reported by others [18, 25–27]. However, 50% of children required addition of MMF but there was no difference in response (91% vs 87%). These figures are consistent with a study comparing CS with MMF and showed that CS is superior to MMF in steroid sensitive nephrotic syndrome [28]. MMF was well tolerated without significant gastritis or BMS in our study as mentioned in the literature.

Considering long term outcome, majority (46%) of children were maintaining CR or PR remission off therapy whereas 35% were maintaining CR/PR on immunosuppressive therapy. This is outcome of cohort of DNS over 12 years which cannot be compared with either SSNS with good long- term prognosis or with SR one which may progress to CKD in more than 40% over 10 years [12].

Strength and limitations of study: Though, the current study comprised of large cohort with good long term follow up (12 years), managed by single nephrologist at a single center but it was retrospective and not comparing the response of ISAs according to histological type. Direct comparison of CPM with levamisole or CS with MMF was not attempted.

Future comparative studies on effectiveness of levamisole, CPM, MMF and CS based on histopathological diagnosis both in FR/SD and SRNS are needed.

## Conclusion

We found that majority of patients were FR/SD among DNS and MCD was most common underlying pathological lesion. Levamisole and CPM were effective in maintaining remission in FR/SD group. FSGS was the most common cause of resistance to steroid and other alternative agents. Though, CSA was effective in inducing remission in SR, but progression to CKD occurred in 11cases. Mortality (2.84%) was mainly due to infectious or uremic complications.

## Abbreviations

AKI: Acute kidney injury
CKD: Chronic kidney disease
CNIs: Calcineurin inhibitors
CPM: Cyclophosphamide
CR: Complete remission
CS: Cyclosporin
DNS: Difficult nephrotic syndrome
FRNS: Frequent relapsing nephrotic syndrome
FSGS: Focal segmental glomerulosclerosis
IgM NP: Immunoglobulin M Nephropathy
ISAs: Immunosuppressive agents
KDIGO: Kidney disease initiative global outcome
LEVA: Levamisole
MCD: Minimal change disease
MGN: Membranous glomerulonephritis
MMF: Mycophenolate mofetil
MPGN: Membranoproliferative glomerulonephritis
OP: Oral prednisolone
PR: Partial remission
SDNS: Steroid dependent nephrotic syndrome
SRNS: Steroid resistant nephrotic syndrome
SSNS: Steroid sensitive nephrotic syndrome
suPCR: Spot urine protein creatinine ratio
Tac: Tacrolimus
